# High throughput screening of starch structures using carbohydrate microarrays

**DOI:** 10.1038/srep30551

**Published:** 2016-07-29

**Authors:** Vanja Tanackovic, Maja Gro Rydahl, Henriette Lodberg Pedersen, Mohammed Saddik Motawia, Shahnoor Sultana Shaik, Maria Dalgaard Mikkelsen, Susanne Langgaard Krunic, Jonatan Ulrik Fangel, William George Tycho Willats, Andreas Blennow

**Affiliations:** 1 Department of Plant and Environmental Sciences, University of Copenhagen, Denmark

## Abstract

In this study we introduce the starch-recognising carbohydrate binding module family 20 (CBM20) from *Aspergillus niger* for screening biological variations in starch molecular structure using high throughput carbohydrate microarray technology. Defined linear, branched and phosphorylated maltooligosaccharides, pure starch samples including a variety of different structures with variations in the amylopectin branching pattern, amylose content and phosphate content, enzymatically modified starches and glycogen were included. Using this technique, different important structures, including amylose content and branching degrees could be differentiated in a high throughput fashion. The screening method was validated using transgenic barley grain analysed during development and subjected to germination. Typically, extreme branching or linearity were detected less than normal starch structures. The method offers the potential for rapidly analysing resistant and slowly digested dietary starches.

Starch is the principal storage polymer in the majority of plants. It accumulates as a complex granular structure made of the glucan homopolymers amylose and amylopectin, organised into large, distinctly shaped insoluble granules. Amylose typically makes up 25–30% of the starch granule, it is an essentially linear molecule that possesses an α-1,4 linkage backbone structure but can be sparsely branched by α-1,6 linkages. Amylose is mostly amorphous in the starch granule. Amylopectin is a highly branched molecule, typically comprises 70–75% of the starch granule, is more than 100-fold larger than amylose, contains clustered α-1,6 linkages which promotes crystalline lamellae to be formed in the granule^1^.

High throughput (HTP) screening of polysaccharide structures is becoming increasingly important, especially in the field of plant breeding to permit fast evaluation of, for example, mutant collections. Since the discovery that starch binds tri- and polyiodide and forms a strong complex with amylose, this method was used as “amylose indicator”, to quantify amylose content in starch. Amylose in the presence of the iodide ligand, changes conformation to left-handed single helix V-amylose which cavity provides space for iodide and results in a bright blue complex^2^,^3^. Starches without amylose make brown-red complex, as iodine binds only weakly to the short helical segments in amylopectin molecules. Spectrophotometric assays were developed based on this complexation^4^,^5^ but most importantly, this technique provides an important HTP screening opportunity and permitted the identification of low amylose potato lines by screening of thousands of lines in a mutant collection^6^. This method is also valuable for identifying starches that resists amylolytic degradation, so called resistant starch or RS. These starches are typically characterised by having high amylose but also includes highly branched starches^7^,^8^,^9^. However, while iodide complexation can indicate information about amylose-amylopectin ratio and is useful for HTP screening of amylose, this method is not quantitative and does not yield any detailed information about the underlying starch structures.

More exact structural information in polysaccharides may be obtained by using the very specific recognition conferred by monoclonal antibodies (mAb) and carbohydrate binding modules (CBMs). Potential starch-binding CBMs are already known, but as often with molecular probes, determining their binding specificity can be a challenging process. However, a HTP method for characterising probes was published recently^10^. The method utilizes carbohydrate microarrays populated with defined oligosaccharides conjugated to BSA. One microarray can contain hundreds of distinct samples and an unknown probe can be screened against all the defined samples on the array in a few hours. Another microarray-based technique, Comprehensive Microarray Polymer Profiling (CoMPP), has also been developed that enables the polysaccharide content of plant samples to be determined. CoMPP is based on the extraction of polysaccharides which are then printed on arrays and probed with mAbs or CBMs to reveal the relative abundance of glycan epitopes across the sample set^11^. However, no starch recognising probes have been reported for this purpose.

In this study we further developed carbohydrate microarray analysis by creating microarrays populated with various starch samples including oligo- and polysaccharides, and cereal grain samples transgenically modified to generate specific starch molecular structures. These arrays were used to determine in detail the recognition profile of CBM20, a known starch-recognising protein module. CBM20 is the first assigned and best described family present in starch-active glycoside hydrolases including glycoamylases, α-amylases, β-amylases, cyclodextrin glucanotransferases and starch-interacting non-amylolytic enzymes like glucan water dikinases^12^. CBM20s exhibit bivalent binding, mediated by two separate glucan-binding sites. These sites have different structures and therefore different functional roles, typically dependent on specific aromatic amino acids with conserved positions, like tryptophan and tyrosine, which stack and interact with glucose residues in the starch granule. A smaller, rigid ‘site 1’ is probably the initial starch-recognition region, while a larger ‘site 2’ guides the starch chain towards the active site and undergoes significant structural changes upon binding^13^. CBM20s main function is to attach to granular starch, but they also bind to smaller maltooligosaccharides such as maltoheptaose and starch-mimicking cyclodextrins^14^. CBMs confer proximity effects for enzymes containing CBMs by binding to the starch to locally increase the substrate concentration at the substrate surface, and even disrupting or “unwinding” α-glucan helices on the granule surface, resulting in a higher hydrolytic rate^15^,^16^.

Glycoamylase from *Aspergillus niger* G1 (GA, 1,4-α-D-glucan glucohydrolase, E.C. 3.2.1.3) contains currently the best characterised CBM20. Glycoamylase consists of two functional domains: N-terminal catalytic domain (residues 1–470, 55 kDa) and a C-terminal CBM 20 (residues 509–616, 12 kDa), which are connected by a heavily O-glycosylated serine/threonine-rich linker. It has defined conserved residues crucial for two sites: binding site 1 contains Trp543 and Trp590; while Tyr527 and Tyr556 are assigned to the binding site 2^15^,^17^. The two binding sites have different affinity towards starch, site one having low affinity^12^. However, onsite affinity calculations range 1.7–200 μM affinity towards the ligand depending on ligand structure and methods used^12^,^14^. Most importantly, the affinity for starch is seemingly very specific^16^,^18^,^19^,^20^, which is important for HTP applications of complex samples. However, the different affinity towards different molecular structures like the presence of α-1,6-branch points and phosphate substitution as well as how affinity is affected by different glucan aggregation including crystalline polymorph and amorphicity is less well-documented.

This paper introduces CBM20 as a specific HTP probe for starch and α-glucan recognition in carbohydrate microarray technology. The potentially different specificities for linear, branched and phosphorylated maltooligosaccharides were evaluated and specificity assessed in comparison with other polysaccharides using specific antibodies as probes and validated using complex transgenic plant samples.

## Results and Discussion

We tested the oligosaccharide and starch microarrays for CBM screening by probing microarrays with CBM20 and four mAb: BS-400-2, BS-400-3, LM6, LM10 and LM11. BS-400-2 and BS-400-3 bind to the β-glucan epitopes, (1–3)-β-glucan and (1–3)(1–4)-β-glucan, respectively^21^. LM6 binds to (1,5)-α-L-arabinan^22^, LM10 is specific to unsubstituted or sparsely-substituted xylans, while LM11 binds to arabinoxylan and unsubstituted xylan^23^.

**Figure 1:**
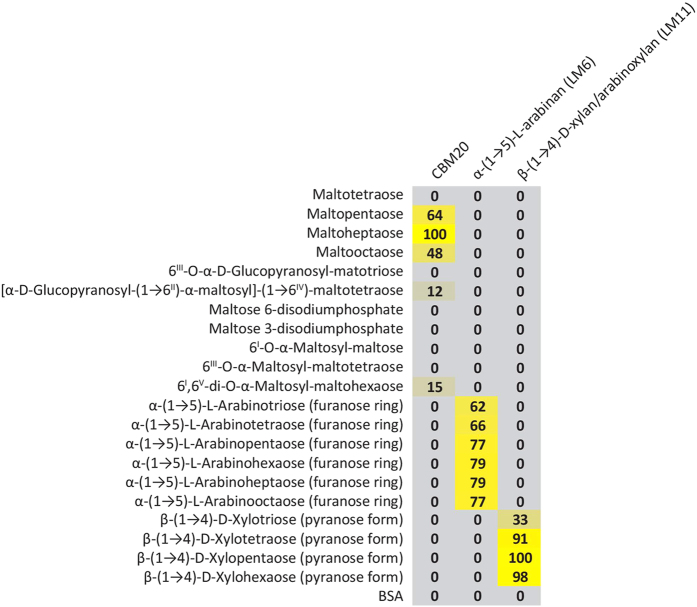
Defined oligosaccharide microarray. Specificity screening of CBMs and mAbs. Defined carbohydrate microarrays populated with a selected set of oligosaccharides including chemically synthesised branched maltooligosaccharides. The mean spot signals are presented as a heat map in which color intensity is correlated to signal with step of 10 for the colours. The highest signal within each type of molecular probe was set to 100, and all other values were normalized accordingly. A low end cut-off value of 5 was used.

CBM20 did not bind to maltooligosaccharides smaller than maltopentaose (Fig. 1). It had an increasing affinity towards the linear maltooligosaccharides pentaose and heptaose. Binding was somewhat weaker towards maltooctaose. Additionally, CBM20 bound weakly to the branched maltodecaoligosaccharide and maltoheptaoligosaccharides 6I,6V-di-O-α-maltosyl-maltohexaose and [α-D-Glucopyranosyl-(1-6II)-α-maltosyl]-(1-6IV) -maltotetraose, indicating that the α-(1–6) branch point restricts binding and that heavily branched maltooligosaccharides are not epitopes for CBM20. Binding was not observed for arabinooligosaccharides and xylooligosaccharides indicating high specificity towards maltooligosaccharides. However, potential xylan-binding capability has been demonstrated for a CBM20 in an amylopullulanase from *Thermoanaerobacter pseudoethanolicus*^24^. Finally, CBM20 did not interact with the phosphorylated maltoses showing that phosphate monoesters do not contribute to binding^14^. It is known that CBM20 binds to the linear maltoheptaose composed of solely α-(1–4) linkages^13^,^15^. Taken together these data indicate that CBM20 has an optimal affinity towards linear maltooligosaccharides of approximately DP7. The decreased binding to the maltooctaose (DP8) as compared to maltoheptaose (DP7) can however be an effect of glucan aggregation on the membrane.

**Figure 2:**
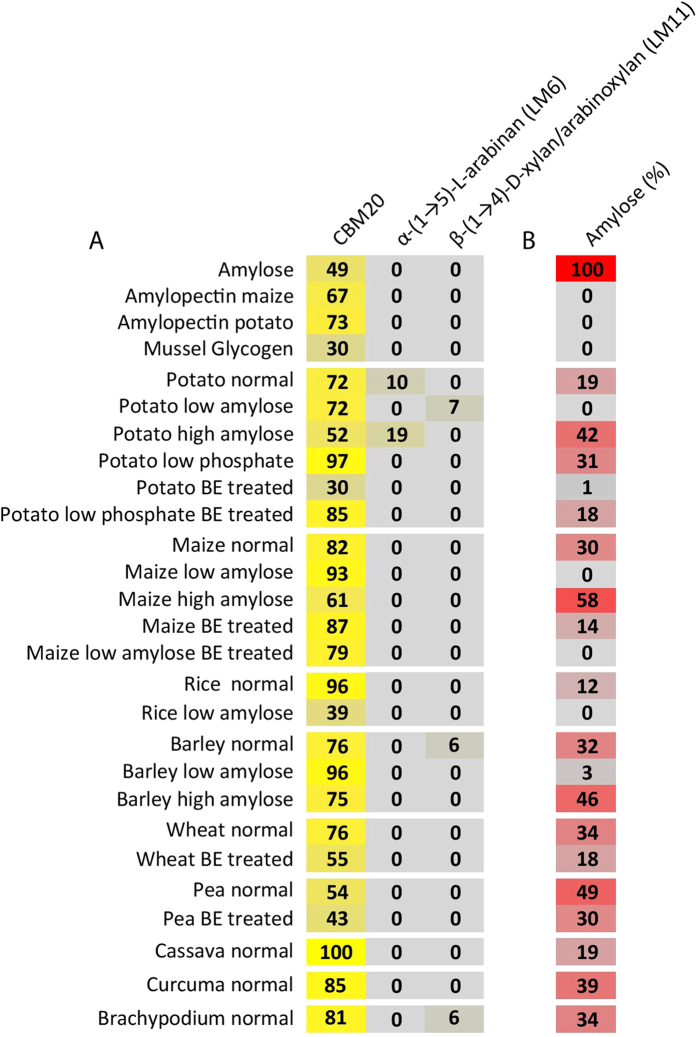
Defined carbohydrate microarray. Specificity screening of CBMs and mAbs (**A**). Defined carbohydrate microarrays populated with a selected set of polysaccharides and probed with CBMs and mAbs. The mean spot signals are presented as a heat map in which color intensity is correlated to signal. Heatmap parameters were set as in Fig. 1. The heatmap (**B**) shows amylose percentages.

CBM20 was used to probe starch samples prepared from different botanical sources and of known amylose content. The binding of CBM20 (Fig. 2A) was not clearly correlated with the amount of amylose (Fig. 2B). This was exemplified by the strongest signal found for CBM20 as observed for cassava starch, which contains 19% amylose whilst potato starch having the same amount of amylose showed much weaker binding. However, from very low to medium amylose concentrations the relative interaction signals increased up to 60% of full signal, after which binding levels stayed at similar high level except for some high amylose samples for which the signal was lower. One likely explanation for these findings is that the determining feature of binding is an unknown substructure(s) of amylose and amylopectin, and starch “fingerprint”-amylopectin chains, for example differently positioned branch points. CBM20 showed very weak binding to glycogen (highly branched) and typically to branching enzyme (BE) treated starch^25^ although these polysaccharide have short linear segments expected to be potent epitopes for CBM20, as judged from the oligosaccharide signals. A possible explanation is that the branches in glycogen (mussel glycogen) are too dense to accommodate interaction with the CBM20, restricting interaction with its two binding sites^13^,^16^. The high amylose starches showed relatively weak binding as compared to the normal starches having approximately 25% amylose. In conclusion, starches with levels of amylopectin and amylose typically found in Nature were generally detected well, whilst highly or loosely branched polysaccharides were detected less. As opposed to iodine complexation screening only detecting maltooligosaccharides larger than approximately DP20^26^,^27^, and if branched even shorter, microarray screening can detect maltooligosaccharides down to at least linear maltopentaose.

**Figure 3:**
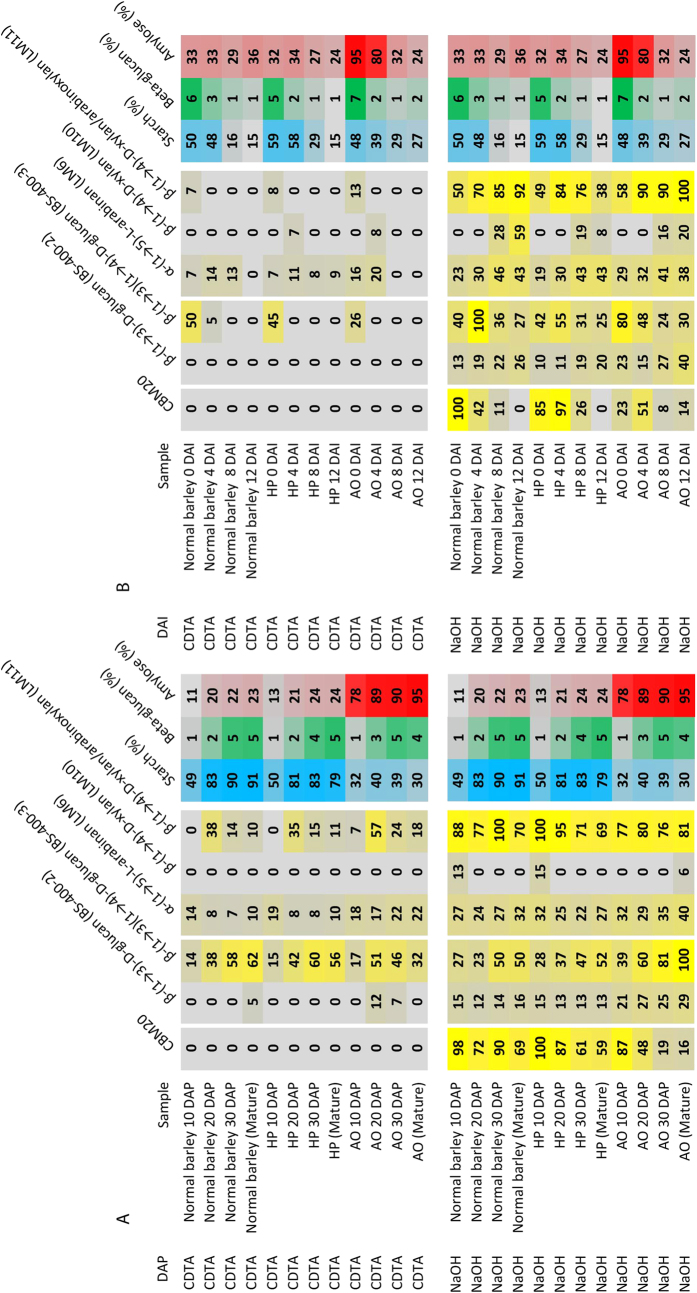
Extracted carbohydrate microarray. Validation of the microarray screening by analysis of polysaccharides in seeds from a diverse set of barley grain samples over development (**A**) and germination (**B**) using CoMPP^11^. The heatmap, in which mean spot signals are correlated with colour intensity, shows the relative abundance of polysaccharide components as extracted using 1,2-diaminocyclohexanetetraacetic acid (CDTA) and NaOH, as well as starch, protein, beta glucan and amylose percentages. Heatmap parameters were set as in Fig. 1. The same amount of cell wall material (alcohol insoluble residue) was used for each sample.

To validate the microarray method using more complex polymeric samples, CBM20 screening was performed using CoMPP arrays populated with extracts from developing and germinating barley grain^28^,^29^. These are transgenic barley lines with RNAi suppressed starch branching enzyme activity resulting in an amylose-only (AO) starch phenotype^30^ and a hyperphosphorylated (HP) starch generated by overexpression of the potato glucan water dikinase (GWD)^31^. The CDTA extraction did not show any detection of starch by CBM20, which was expected as CDTA primarily solubilise highly soluble and calcium immobilised polysaccharide components like pectin. On the other hand the NaOH extraction, which normally extracts hemicelluloses, seemed adequate for extracting starches, as seen by the strong signals for CBM20 when used to probe NaOH-extracted samples^11^ (Fig. 3).

Grain samples for the barley control collected during grain development showed strong CBM20 constant binding while the corresponding HP and AO lines showed decreased binding with development of the grain. This effect was especially evident for the AO line (Fig. 3A). The AO samples showed less binding than the normal barley and the HP at similar starch contents demonstrating that the array signals did not correlate with the starch content *per se* but to specific molecular structures in the grain. (Fig. 3A). As guided by the interactions found for the starch models (Fig. 2) CBM20 possibly detects amylopectin and not so efficiently amylose making CBM20 signal sensitive to amylose content of the starch suppressing the signal as compared to the total starch content in the grain sample. This is in agreement with the low CBM20 affinity to linear (amylosic) maltooligoaccharides (Fig. 1). A trend of LM11 (recognising arabinoxylan and unsubstituted xylan) binding was observed for the CDTA extracted samples showing highest signals at 20 days after pollination (DAP) for all the three normal barley, AO and HP lines indicating the presence of CTDA-soluble transient structures of arabinoxylan since the LM6 (xylan recognising antibody) did not recognise any material. NaOH extracted significantly more arabinoxylan in all samples. The BS-400-3 antibody recognising (1–3)(1–4)-β-glucan steadily increased over development, both for the CTDA and the NAOH solubilised samples demonstrating that this detection is semi-quantitative both for the barley grain water-soluble and non-soluble (NaOH)betaglucans. (1–3)-β-glucan was detected at very low signals in all samples but was highest, and increasing over development, in the AO samples.

Microarrays populated with extracts from grain during germination showed that CTDA principally extracted (1– > 3,1– > 4)-beta-glucan from the mature grain samples. The samples sequentially extracted with NaOH contained also other types of more recalcitrant polysaccharides, including starch. CBM20 interaction was strongest to the mature starch from the normal barley and HP from grain at the very beginning of imbibition (Fig. 3B) followed by a dramatic decrease. The AO signals for CBM20, just as for the development samples, were generally low. However, moderate binding was observed for 4 days after imbibition (DAI) indicating a weakening of the starch structures as a result of amylolytic degradation in the grain. The signals for all three starches weakened towards 12 DAI. This was due to the very low grain starch contents at later stages (Fig. 3B). However, again the array signals did not fully correlate with the starch content (Fig. 3B) demonstrating the different structures developing over germination. High phosphorylation did not markedly impact binding profiles.

## Conclusions

Our data provides valuable insight into the specificity of CBM20 and its use as a specific probe for starch. Based on its differential recognition, CBM20 presents a promising probe for discriminating specific starch structures, preferably chain-lengths found in normal starch types. For starch quantification, the method requires a combination with non-discriminating data for different starch structures and such probes are yet to be developed. In that case, CBM20 can serve as valuable probe for detecting aberrant structures of starches that resists degradation, so called resistant starch including high amylose and highly branched starches.

## Material and Methods

### Starches and maltooligosaccharides samples

Maltooligosaccharides, pure potato amylose and amylopectin were purchased for Sigma. Branched and phosphorylated maltooligosaccharides were synthesised^32^,^33^,^34^,^35^,^36^. Transgenic potato starches of various structures were collected and purified according to^37^,^38^. Starches were purchased accordingly: maize starch (Cerestar-AKV, Iceland), normal potato starch (Andelskartoffelmelsfabrikken, Denmark), cassava, pea, rice (Kartoffelmelcentralen, Denmark), barley (SW Seed AB, Sweden), and *Curcuma zedoaria* (Rohokeimeida, Japan). Starches modified with branching enzyme were from^25^. Normal barley starch of the cultivar Golden Promise and transgenic amylose-only (AO) and hyperphosphorylated barley starch (HP) in Golden Promise genetic background were prepared as described in^30^,^31^. Transgenic potato lines have been developed as described^39^. Branched alfa-glucan purity was validated by ^1^H- and ^13^C-NMR and exceeded 97%. No additional sugar structures could be identified. For the commercial glycans, purity was higher than 95% and mainly contaminated with hydrolysis products as assessed by HPAEC-PAD analysis. Purity of native starches was higher than 95% slightly contaminated with trace cell wall residue and protein.

### Amylose, starch and betaglucan analysis

The apparent amylose content in purified starch was determined by iodide colorimetry as described^40^. Starch and betaglucan in barley grain material were analysed as described^37^,^38^.

### Microarray printing, microarray probing and analysis

**Figure 4:**
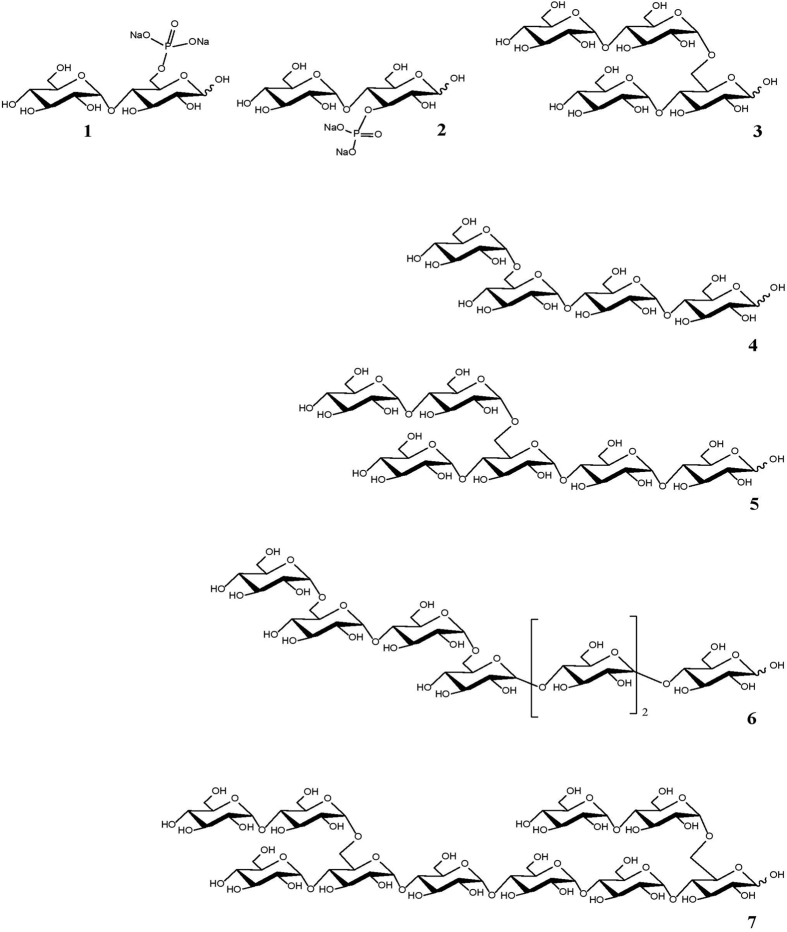
Structures of the branched and phosphorylated maltooligosaccharides. 1: maltose 6-disodiumphosphate; 2: maltose 3-disodiumphosphate; 3: 6I-O-α-maltosyl-maltose; 4: 6III-O-α-D-glucopyranosyl-matotriose; 5: [α-D-glucopyranosyl-(1-6II)-α-maltosyl]-(1-6IV)-maltotetraose; 6: 6III-O-α-maltosyl-maltotetraose and 7: 6I,6V-di-O-α-maltosyl-maltohexaose.

**Figure 5:**
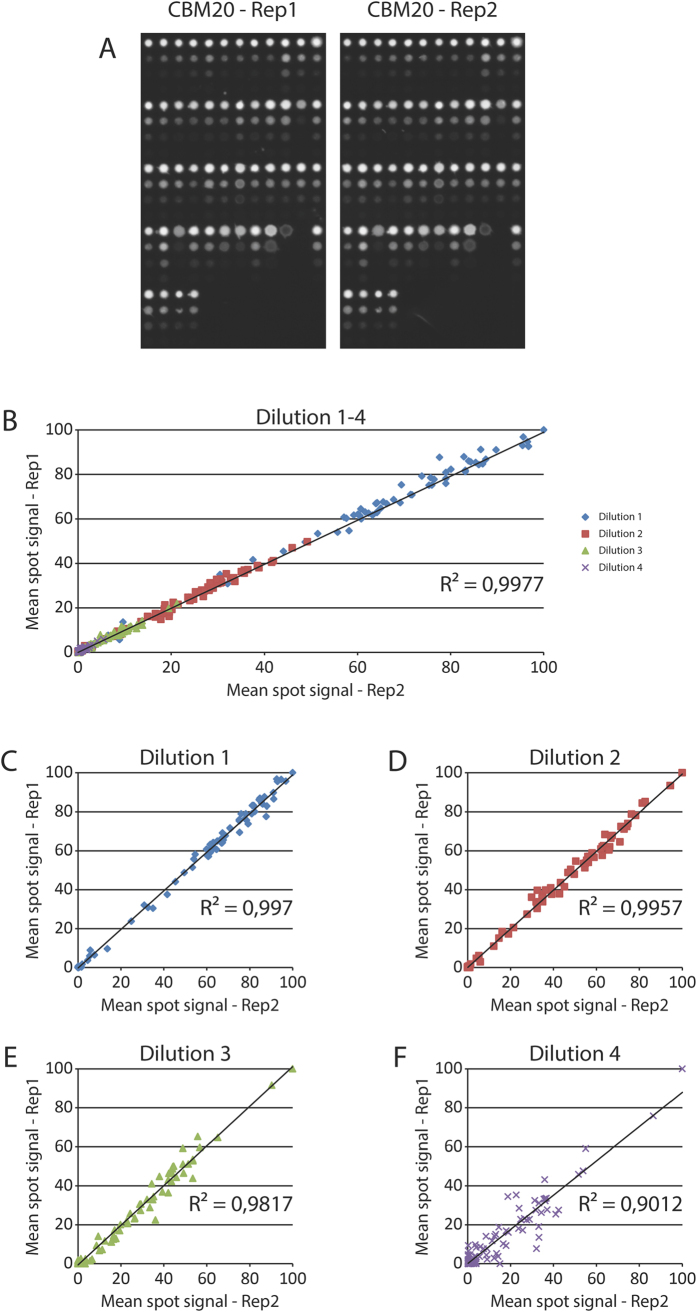
Print replicates, morphology, quality and reproducibility for arrays probed with CBM20. (**A**) Array probed with CBM20 showing print replicate 1 and 2 showing microarray spot morphology, quality and reproducibility. (**B**) The replicates plotted against each other with r2 values. (**C–F**) The individual dilutions plotted against each other. Reproducibility falls off for the fourth dilution due to low or no of signal. Axes on the graphs are relative mean spot signal.

Defined and extracted glycan microarrays were printed as described^10^,^11^. Structural information on the branched and phosphorylated maltooligosaccharides are depicted in Fig. 4 and in Fig. 5 spot morphology, reproducibility and quality are documented. Briefly the arrays where printed using an Arrayjet Sprint (Arrayjet, Roslin, UK) utilising piezoelectric technology. They were spotted on nitrocellulose membrane with a pore size of 0.45 μm (Whatman, Maidstone, UK) under conditions of 19 °C at 55% humidity. All samples were diluted in buffer containing 55.2% glycerol, 44% water, 0.8% Triton X-100 and printed with three 5-fold dilutions to a total of 4 spots, each with a technical replicate. Oligosaccharides were diluted to a 2 mg/ml and a 0.04 mg/ml concentration in deionised water immediately before use and transferred to a 96-well microplate for printing. The arrays were blocked in PBS (phosphate buffered saline) containing 5% v/w low fat milk powder (MPBS). Following, the arrays were washed in PBS and probed for 2 h with CBM20 or primary antibodies (0.02 mg/ml) in MPBS. In the subsequent step the arrays were washed with PBS and incubated for 2 h with anti-mouse or anti-rat secondary antibodies conjugated to alkaline phosphatase (Sigma, Poole, UK) diluted 1/5000 in 5% MPBS before developed using 5-bromo-4-chloro-3-indolylphosphate and nitro blue tetrazolium in alkaline phosphatase buffer. Microarrays were scanned at 2400 dpi using a flatbed scanner (Cannon, Søborg, Denmark) and converted to grayscale TIFFs. Scanned microarrays were analysed using Array-Pro Analyser (MediaCybernetics, USA) and presented as heat maps in which colour intensity was correlated to mean spot signals.

### CBM20 cloning expression and purification

The CBM20 encoding gene fragment from *Aspergillus niger* (327 bp)^41^ was synthetically made and subcloned into pET-28a(+) between NcoI and HindIII cloning sites by Invitrogen, Life Technologies Corporation. The resulting construct is CBM20 with 6xHis. The plasmid was amplified in *Escherichia coli* strain DH5α (F– Φ80lacZΔM15 Δ(lacZYA-argF) U169 recA1 endA1 hsdR17 (rK–, mK+) phoA supE44 λ– thi-1 gyrA96 relA1)^42^. Protein was expressed in *E. coli* BL21(DE3) (F– ompT hsdSB(rB–, mB–) gal dcm (DE3)) in LB medium; containing 50 μg/ml kanamycin, at 37 °C, at 250 rpm, to OD_600_ = 0.6. Expression was induced by adding isopropyl β-D-thiogalactopyranoside (IPTG) to 1 μl/ml, followed by incubation at 20 °C for 20 h. The CBM20 probe was purified using a combined HisTrap and β-cyclodextrin chromatography procedure (Christiansen *et al.*^14^). Sample was applied with a flow of 0.5 ml/min on a 1 ml HisTrap column (GE Healthcare). And the non-bound protein eluted with 20 column volumes of washing buffer (20 mM HEPES (4-(2-hydroxyethyl)-1 piperazineethanesulfonic acid), 20 mM imidazole, 500 mM NaCl, 5% glycerol pH 7.4). CBM20 protein was eluted with 20 column volumes of elution buffer (20 mM HEPES, 500 mM imidazole, 500 mM NaCl, 5% glycerol). Eluted protein was applied to a 6 ml β-cyclodextrin (β-CD) linked Sepharose column using a 0.5 ml/min flow. Bound protein was washed with 2 column volumes 50 mM NaCl, 250 mM NaCl pH 7.5 and eluted in 50 mM HEPES, 10 mM β-CD, pH 7.5. Eluted fractions were analysed by sodium dodecyl sulfate polyacrylamide gel electrophoresis (SDS-PAGE) to detect CBM20 protein and to verify electrophoretic purity. Relevant fractions combined for dialysis (mMw cut-off, MWCO 3.5) against 50 mM HEPES, pH 7.5, 10% glycerol. The CBM20 protein was stored at −20 °C until used. The CBM20 module has previously been shown to be robust (Christiansen *et al.*^14^).

### Antibody probes

Two antibodies were used with specificity towards glucan epitopes: BS-400-2 and BS-400-3 bind (1–3)-β-glucan and (1–3)(1–4)-β-glucan, respectively^21^. These mAbs were obtained from Biosupplies Australia. mAb LM6 binds to (1,5)-α-l-arabinan^22^. LM10 is specific to unsubstituted or sparsely-substituted xylans, while LM11 binds to arabinoxylan and unsubstituted xylan^23^. LM6, LM10 and LM11 were obtained from PlantProbes, UK.

## Additional Information

**How to cite this article**: Tanackovic, V. *et al.* High throughput screening of starch structures using carbohydrate microarrays. *Sci. Rep.* **6**, 30551; doi: 10.1038/srep30551 (2016).
